# Supplementary material for: Are carbon emissions trading and green financial instruments synergistic? -Comprehensive quantitative research based on content analysis
Source: PLoS One. 2024 Mar 7;19(3):e0298601. doi: 10.1371/journal.pone.0298601 (PMC10919601; doi:10.1371/journal.pone.0298601)
Supplement: S1 Data — (ZIP) [file pone.0298601.s001.zip › Data/地级市Z_F工作报告.zip!/░▓╗╒/░▓╟∞╩╨/└┤╫╘╣1⁄2╓┌║┼í╛┬φ┐╦╩2╛▌═°í┐_2012.docx]

**政府工作报告**

——2012年1月31日在安庆市第十五届

人民代表大会第四次会议上

市长  肖超英

各位代表：

现在，我代表市人民政府，向大会报告政府工作，请予审议，并请市政协委员和其他列席人员提出意见。

一、2011年工作回顾

    过去的2011年，是实施“十二五”规划第一年。全市人民在省委省政府和市委的坚强领导下，深入贯彻落实科学发展观，加快经济发展方式转变，顺利完成市十五届人大三次会议确定的年度目标任务，实现了“十二五”良好开局。预计全市生产总值1200亿元，同比增长13%以上；财政一般预算收入156.7亿元，增长29.4%；全社会固定资产投资830.2亿元，增长27.9%；社会消费品零售总额397.5亿元，增长17.5%；进出口总额8.2亿美元，增长25%；城镇居民人均可支配收入17400元，增长17%，农民人均纯收入5900元，增长18%；城镇登记失业率控制在4.2％以内；人口自然增长率为4.9‰。

**（一）有效需求不断扩大。**千方百计增投入、扩内需、稳外需。省“861”重点项目完成投资215.3亿元，市“3231”重点项目完成投资316.2亿元。争取中央和省补助资金16.64亿元，带动地方投资35.74亿元。安庆长江铁路大桥等项目加快建设，望东长江公路大桥、岳武高速公路安徽段等开工建设。积极培育消费热点，家电下乡和以旧换新工作居全省前列。大力促进机电和高新技术产品出口。加快地方金融体系建设，农信社改制基本完成，新设立村镇银行4家、小额贷款公司15家。全市金融机构存款余额1408.6亿元，总量、增量均居全省第3 位；贷款余额694.3亿元，新增贷款143.3亿元，居全省第4位，创历史最好水平。

**（二）承接产业转移势头强劲。**编制出台“6+2”重点产业发展专项规划。出台实施园区带动战略若干意见。推进园区合作共建，望江县与绍兴共建桥港经济开发区，怀宁县与浙江共建诸暨机械工业园，宿松县与浙江瑞唐公司共建浙商产业园。园区工业总产值达730亿元，增长40%。桐城双新开发区、宿松临江产业园加快建设。市产业集中区（化工区）基本形成凤凰片10平方公里骨干路网。突出集群承接，狠抓招大引强，引进50亿元以上重大项目1个，当年新签约并履约5亿元以上项目32个、亿元以上114个，当年在建投产和新开工亿元以上项目149个。上海华谊集团总投资100亿元化工新材料基地项目落户，总投资40亿元安踏工业园项目开工建设，与中石化共同投资10亿元丁辛醇项目加快推进。实际利用市外资金717.6亿元，增长22.3%；利用境外资金2.72亿美元，增长15.76%。

**（三）工业转型升级步伐加快。**制订公布产业目录，落实结构性减税政策，发挥财政资金引导作用，加速壮大优势产业，改造提升传统产业，积极培育战略性新兴产业。规模以上工业企业1145家，实现增加值455.1亿元,增长21.2%。全市新增私营企业1900家。石油化工、纺织服装、机械与装备制造业产值分别达到375.9亿元、252.6亿元、220.5亿元，增长22.9%、57.7%、49.1%。林浆纸一体化等项目建成，800万吨石化炼化一体化、怀宁上峰水泥二期、华茂纺织工业城加快建设。安庆中船与世界500强美国卡特彼勒公司合资生产马克柴油机项目顺利签约。新增省级高新技术和创新型企业12家，发明专利授权量增长143.8%。开展首届质量奖评选活动。华茂集团创立国家企业技术中心。和兴化工生化合成技术列入国家“863”计划。建立5亿元战略性新兴产业发展引导资金。中船动力、海康通讯等一批高新技术项目投产运营，非晶软磁设备、安簧活塞生产线等项目加快建设。

**（四）节能减排成效明显。**对饮用水源保护区、重金属、化工、造纸企业开展环境隐患安全大排查、大整改，建立巡查、挂牌督办、约谈、责任追究等监管制度。共排查企业985家，停产整改54家，关闭17家。加强建设项目环保验收和“三同时”环境监管。强化规划环评，严把新建项目环境准入关。加强对重点领域、重点行业和关键环节监管，基本完成化学需氧量、氨氮、二氧化硫、氮氧化物减排总量任务。8个县（市）城镇污水处理厂全部建成并投入运行。市区日处理600吨生活垃圾焚烧发电厂和日处理30吨医疗废弃物集中处置中心建成运营，国家级区域性大型再生资源回收利用基地二期工程加快建设。加大重点行业工程减排投入，重点企业污水处理厂加快建设。

**（五）新农村建设取得实效。**粮食总产达 267.6万吨，获全省粮食生产先进市。落实强农惠农政策，一卡通发放补贴资金17.65亿元。畜牧业、水产业规模养殖比重提高3.5个百分点。桐城、怀宁现代农业示范区加快建设。启动实施农业产业化转型倍增计划，龙头企业产值达374亿元，增长22%，国家级龙头企业达6家，省级龙头企业69家，各类农业产业化组织发展到1900多个。推进水利基础设施建设，除险加固中小型水库55座，解决32.24万农村人口饮水安全问题。实施千村百镇示范工程，省级示范村、镇达200个和21个。改造县乡公路123.6公里。严格耕地保护，耕地保有量613.5万亩，新增耕地2.2万亩。规范土地流转，耕地规模经营比重达28.5%，居全省前列。集体林权制度配套改革扎实推进，新增营造林15.1万亩。

**（六）新型城镇化扎实推进。**新一轮城市总体规划获省批准。获批建设用地1.7万亩，收储土地1万余亩，出让土地合同价款64亿元。市政务、商务、体育中心建设加快推进。顺安路二期、振风大道二期、人民路延伸段、华亭大沟区域清淤、集贤关山体整治一期等续建工程基本完工，钱牌楼街、工农街中段等10条老城区小街小巷道路改造完工。人防工程建设突破10万平方米。启动创建国家森林城市工作，城区新增47万株苗木栽植。成功创建安徽省文明城市。桐城、太湖获省级文明县城。重点建设项目快速推进，桐城龙眠河整治工程完工，枞阳莲花湖公园建成开放。完成130个村庄整治，改造1.7万户农村危房。累计投入4.78亿元升级改造农村电网。宜秀区杨桥地震灾后重建17万平方米小区主体工程完工。引导非农产业、农村人口有序向城镇转移，城镇化率达43.9%，提高1.9个百分点。

**(七)文化旅游融合发展。**天柱山风景区成功创建国家5A景区，荣获世界地质公园称号。太湖五千年文博园建成开园，成为全国文化旅游产业示范基地。再芬黄梅股份有限公司挂牌成立。亿元以上在建旅游项目达116个。孔雀东南飞影视基地、石莲洞、浮山、明堂山、天峡创建4A景区获批。孔城老街、枞阳浮山试开放，岳西彩虹瀑布基本建成，天柱山养生中心、中国桐城派文化博物馆、花亭湖旅游码头等项目进展顺利。安庆港实现与台湾直航。全年接待海内外游客2312万人次，增长28.3%；旅游总收入159.5亿元，增长31.8%。

**(八)人民生活持续改善。**出台实施价格调节基金征管办法，完善社会救助和保障标准与物价上涨挂钩联动机制，及时发放城区困难群众价格补贴，强化生猪和蔬菜市场供应，实行肉价临时补贴。出台推进新一轮“菜篮子”工程建设意见，新建城郊蔬菜基地4295亩。大观区海口镇加强“菜园子”建设，中心城区加强“菜市场”建设和管理。居民消费价格指数增长5.5%，低于全省水平。42项民生工程投入资金55.83亿元。城镇新增就业5.18万人，帮扶下岗失业人员再就业2.39万人，农村富余劳动力新增转移就业6万人。加快完善社会保障体系，基本养老、基本医疗、失业保险参保人数分别达到52.07万、39.33万和25.9万，新农合参保人数488.18万，参合率98.4%。累计农村五保供养床位2.3万张，供养率31%。提高失地农民养老金补助标准。加强保障性住房建设，开工2.4万套，建成1.4万套。开展扶贫新一轮整村推进工程，减少贫困人口7万人，扶贫开发工作获全国先进。高度重视校园安全，校安工程投入资金8.62亿元，加固改造校舍131.6万平方米。新建农村户用沼气8300户。新建乡镇综合文化站42个、农家书屋609个，2136个自然村实现广播电视村村通。

**（九）社会事业加快发展。**全民健身运动蓬勃开展，市首届妇女运动会成功举办。我市荣获全国听力语言康复工作基层示范单位称号。健全医疗纠纷和交通事故调处机制。安全生产形势基本平稳。加强食品药品安全监管。加强社会管理创新，城乡社区功能不断完善。依法打击各类违法犯罪，有效处置突发性事件，社会治安持续稳定，人民群众安全感进一步提升。教育事业稳步发展，安徽黄梅戏学校升为艺术职业学院。卫生工作积极进展，基层医改取得实效。持续加强人口和计划生育工作。国防动员、人民防空、国家安全、双拥优抚和民族宗教工作进一步加强。外事、侨务、对台、统计、档案、地方志、地震、气象、妇女、儿童、老年人事业取得新的进步。

 (**十)政府自身建设不断加强。**坚持把建设人民满意政府放在重中之重，政府服务和管理水平有了新提高。自觉接受市人大及其常委会法律监督和市政协民主监督，进一步密切与工青妇等人民团体的联系，主动加强与各民主党派、工商联、无党派人士的对口联系。办理人大代表建议147件、政协提案523件，办复率100%。“六五”普法全面启动，依法行政深入推进。政务公开不断深化，全年依法公开政府信息5.4万条，清理市政府规范性文件187件，废止、修改81件。深入开展大走访、带案下访活动。健全完善网络问政机制，认真办理网友留言回复。全面强化应急管理机制。进一步规范招投标市场。认真落实党风廉政建设责任制，着力加大重点领域、关键环节的行政监察和审计监督，查处违法违纪案件144件，坚决纠正损害群众利益的不正之风。

 各位代表！

 回顾一年来的工作，成绩来之不易。这是省委省政府和市委正确领导、科学决策的结果，是全市人民同心同德、奋力开拓的结果。在此，我谨代表市人民政府，向全市广大工人、农民、知识分子、干部，向驻宜解放军指战员、武警部队、消防官兵和政法干警，向各民主党派、工商联、无党派人士、各人民团体和社会各界人士，致以崇高的敬意！向关心支持安庆发展的中央和省驻宜单位，向在宜的所有投资者和建设者，表示诚挚的感谢！

在充分肯定成绩的同时，我们也清醒地看到困难和问题，主要是:经济结构不优，大项目大企业少，新兴产业和现代服务业发展基础薄弱；自主创新能力不强，环境保护压力加大；资金土地等要素制约趋紧，中小微企业生产经营困难增多；城市管理体制机制法制不够完善，一些薄弱环节的整治还不到位；有些部门服务意识不强，工作中扯皮推诿、行政不作为、乱作为等现象时有发生。我们必须认真负责地对待这些问题，不回避、不推卸、不护短，尽最大努力切实加以解决。

二、着力抓好2012年各项工作

2012年，是实施“十二五”规划承上启下的重要一年。我们既要看到，当前全球经济复苏的不稳定性不确定性上升，我国经济发展中“两难”问题增多，保持经济平稳较快发展面临新的挑战，总体形势仍将十分严峻复杂；也要看到，经济全球化和区域经济一体化持续深入，产业转移势头强劲，中部地区加速崛起，皖江示范区提速建设；更要看到，我市工业化、城镇化和农业现代化进程加速，经过多年来持续抓项目、增投入、快发展，经济发展的内生动力加速释放。特别是市第十次党代会描绘了建设“四个安庆”的宏伟蓝图，为做好今年乃至今后一个时期工作指明了方向。我们一定要正确把握形势，化挑战为机遇，变压力为动力。

今年工作的总体要求是：**以邓小平理论和“三个代表”重要思想为指导，全面贯彻中央和全省经济工作会议精神，围绕科学发展主题和全面转型、加速崛起、富民强市主线，把握稳中求进、好中求快的工作总基调，着力稳定经济增长，着力调整经济结构，着力深化改革开放，着力保障改善民生，保持经济又好又快发展，保持社会大局和谐稳定，为全面建设“四个安庆”、基本建成全面小康社会奠定坚实基础，以优异成绩迎接党的十八大胜利召开。**

综合考虑，今年全市经济社会发展的主要目标是：地区生产总值增长12%以上，财政一般预算收入增长15%，社会消费品零售总额增长17%，固定资产投资增长25%，城镇居民人均可支配收入增长15%，农民人均纯收入增长17%，居民消费价格涨幅控制在4%左右，城镇新增就业5万人以上，城镇登记失业率控制在4.2%以内，人口自然增长率控制在6.5‰以内，节能减排指标完成省下达任务。

实现上述目标，必须着力抓好以下十个方面工作：

**（一）持续加大投入力度**

保持经济平稳较快发展，是今年的首要任务。必须牢牢把握扩大内需这一战略基点，密切关注宏观环境变化，强化有效投入，增强经济增长的内生动力。

**狠抓项目建设。**加强重点项目调度，全力抓好项目落地和竣工投产。围绕战略性新兴产业、装备制造业和现代服务业等领域，谋划建设一批重大产业项目。加快建设宁安城际铁路、石化炼化一体化、望东长江公路大桥及接线、岳武高速等项目，开工建设皖江电厂二期。大力推进百万吨乙烯、济祁高速安庆段、枞阳至池州长江公路大桥、北沿江高速、阜景铁路、合安城际铁路、民航新机场等项目前期工作。激活民间资本，促进社会投资稳步增长。

**强化金融支持。**引导金融机构加大信贷投入，保持信贷增长高于全省平均水平。优化信贷结构，加强对重大项目、重点企业、中小微企业、“三农”和民生工程倾斜。举办第十届银企信贷项目洽谈会。支持市城投公司发行企业债券20亿元；深化与开行合作，融集保障性住房建设资金12亿元。支持市开发区发行6亿元企业债券。新增2家企业进入上市辅导期。深化农信社改革，培育发展村镇银行等新型农村金融机构，新设小额贷款公司5家以上、融资性担保公司2家。力争引进2家股份制商业银行来安庆设立分支机构。加强金融生态环境建设。

**坚持强势招商。**加大利用境外和市外资金力度。加强驻外招商，遴选干部组建市直驻外招商小组，实行招商项目全程代理制。组织参与徽商大会、中博会、投洽会等重大招商活动。突出产业招商，在京、苏、浙、沪等地举办专题产业推介会。积极参加皖粤合作活动。加强督查调度，强化安商服务，促进项目落地。着力引进总投资50亿元以上项目1个、5亿元以上20个、亿元以上100个，新开工和建成投产亿元以上项目100个。

**（二）坚持做好“三农”工作**

    “三农”工作始终处于重中之重的位置。必须大力提高农业综合生产能力，提升农业现代化水平，加快农民增收步伐。

**加强水利项目建设。**抓住国家加快水利建设机遇，谋划推进一批重大项目。加强农田水利建设，完成高标准农田8万亩，新增高效节水灌溉工程1.5万亩。全力推进下浒山水库、华阳河分蓄洪区安全建设，加快大沙河、皖河流域治理。抓好抗旱设施建设，加快实施大中型灌区续建配套和节水改造，再解决38万农村人口饮水安全问题。开工80座病险水库除险加固、7条中小河流治理、2个山洪地质灾害防治等工程。突出平安水利、资源水利和生态水利，提升水资源综合保障能力。

**推进农业科技创新。**继续实施新增粮食生产能力规划，大力创建高产高效粮食生产示范区和万亩吨粮田示范县。加快实施农业特色板块经济发展规划，着力打造农业特色产业集群。深入实施畜牧业升级计划和水产跨越工程，扶持标准化规模养殖示范场和示范县建设，加强农产品质量安全监控和重大动物疫病防控。扎实开展“农业科技创新年”活动，推进农业科技体制创新。加强基层农技推广、畜牧技术推广、农产品质量监管体系建设。落实“菜篮子”市长负责制，以发展设施蔬菜为重点，深入实施蔬菜产业提升行动。加快实施农业机械化推进工程。积极开展林木、大棚蔬菜、生猪等特色农业保险。

**扎实推进新农村建设。**推广潜山、岳西经验，深入实施农村危房改造和村庄整治，完成7000户农村危房改造任务。推进32个乡镇农村清洁工程建设，建立村收集、乡镇转运、集中处理的农村生活垃圾处理模式和卫生保洁制度。改造升级县乡公路300公里，建设通村公路延伸260公里，完成危桥加固改造210座。

**大力促进农民增收。**实施促进农民收入倍增计划。完成农村土地承包经营权登记试点，盘活农村集体资产。推进土地流转和规模经营。落实惠农补贴政策，加强农民负担监管。深入实施农业产业化转型倍增计划，提升龙头企业带动能力。大力发展农民专业合作社。精心开展农村资金互助试点。深入开展职业农民和农村劳动力转移培训，设立返乡农民工创业投资引导资金，实现农民工创业园县区全覆盖，鼓励农民就地就近创业就业。

**切实加强扶贫开发工作。**实施新10年扶贫开发纲要，执行新的扶贫标准和政策。扎实推进开发式扶贫，构建专项扶贫、行业扶贫和社会扶贫格局。抓好大别山革命老区连片特困地区开发，加快国家扶贫开发重点县贫困乡村发展。完成100个村整村推进工程，加强贫困村基础设施建设，改善生产生活生态条件。促进产业化扶贫，扶持发展产业化扶贫龙头企业。实施雨露培训计划，促进贫困群众就业，增加贫困人口收入。实行扶贫与农村低保两项制度衔接，再减少贫困人口10万人。

**（三）着力提升新型工业化水平**

    加快新型工业化进程是产业结构调整的重要引擎。必须牢牢把握发展实体经济这一坚实基础，推进新兴产业规模化、传统产业现代化，全面提升产业素质和核心竞争力。

**培育发展战略性新兴产业。**实施战略性新兴产业发展规划，围绕新能源、新材料、节能环保、高端装备制造、电子信息等谋划储备一批重点项目。加快建设省新型工业化产业示范基地。大力实施中船马克柴油机、西锐重工等离子和激光切割机、盛运环保设备、和兴化工、海康通讯、风力发电、矿井安全救生设施等一批新兴产业重点项目。

**着力打造千亿产业。**实施石油化工、纺织服装、机械与装备制造业发展规划，打造“十亿项目、百亿企业、千亿产业”。运用先进适用技术改造提升传统产业。加快企业技术改造，实施30个亿元以上重点技术改造项目和列入省调度的45个技术创新项目。大力发展石化炼化下游产品配套项目，推进曙光升级改造，加快华谊新材料、泰发能源科技项目建设。加快建设华茂纺织工业城、安踏工业园。发展壮大汽车零部件、船舶制造产业，积极培育数控机床、输送机械、电工电器、石化通用机械和环保机械等装备制造产业。

**倾力扶持中小微企业发展。**落实各项政策措施，切实减轻企业负担。围绕特色产业和龙头企业，发展配套服务，延长产业链，推进企业集聚，壮大市场主体。建立产业、融资、技术创新、人才培训等公共服务平台，着力破解用工难、用地难、融资难等问题。加强企业资金链风险防范。强化企业家职业素质培训。大力培育“专、精、特、新”企业，提质扩量，新增规模以上企业100家以上。

**加快信息化工程建设。**推进数字城市、无线城市和智慧安庆建设。促进信息化和工业化深度融合，推进信息技术融入企业研发、过程控制、企业管理、市场营销、技术改造、节能减排等环节，实现实时监测和调控，提高生产精确度、工作效率和资源利用率。发展电子商务，提高企业网络采购和销售水平。加快信息产业发展，重点推进汽车电子、船舶电子、机床电子等产品研发。

**（四）不断加快现代城镇体系建设**

    城镇化对加快发展具有重要的驱动作用。必须加快新型城镇化进程，构建中心城市、特色县城、风情小镇、秀美乡村“四位一体”的新型城镇体系。提高城镇化率2个百分点。

**加快中心城市建设。**以新一轮城市总体规划为引领，加快构筑“大宜城”，建设皖西南区域中心城市。完成基础设施投资100亿元以上。加快市政务中心、体育中心、商务中心和金融中心建设，推进东部新城、北部新城、大观新城组团式发展和城市基础设施贯通，启动规划建设市区通往怀宁、枞阳的快速通道。加快环城南路建设，完成环城西线，开工建设潜江路、柘山路二期、文苑路、宜秀大道、机场大道延伸线、振风大道与高速公路连接线。加强大龙山高速出入口综合整治。推进城区主干道路面黑色化。加大力度建设国家农发行贷款40亿元新河综合整治项目以及顺安河、顺安路泵站工程，深入推进花亭南北村内涝综合治理。开工建设城东污水处理厂二期工程，实施污水泵站提升工程。启动一水厂异地扩建。实施人民路、湖心路、老城区小街小巷改造。完善消防基础设施，建设化工区消防站。积极创建国家森林城市。

**推进县城和中心镇建设。**按照城市的标准规划建设县城，加强县城道路、给排水、燃气、园林绿化、环卫、防灾减灾等基础设施建设。加快体育场馆建设。强化规划，完善小城镇基础设施，促进人口向城镇流动，带动农村发展。推进省级扩权强镇试点镇建设，继续开展“宜城杯”小城镇建设竞赛活动，提升城镇建设质量和水平。推动每个县（市）区建设2-3个风情小镇。推进1640个村庄环境整治，着力建设一批秀美乡村。

**加强城镇管理。**创新城市管理，推进城市管理重心下移，强化区级政府城市管理职能。加强县城、城镇的城市管理执法，推进“城管进社区”，建立精简高效、分工合理、职责明确的城镇管理新体制，提高城镇管理效能。加强城市道路管理，提高市政公用设施维护质量。坚决制止、有效遏制违法违章建设行为。严格建筑渣土处置、户外经营和户外广告管理。加强临时摊点设置管理。建立卫生保洁等精细化管理长效机制。健全部门联动机制，加大治乱保畅力度，强化交通秩序整治。营造方便快捷的交通环境、干净整洁的市容环境和舒适宜人的生活环境。

**（五）扎实推进服务业大发展**

推动服务业发展，是扩大需求、提升综合竞争力的重要举措。必须强化政策支持，建立1亿元服务业发展引导资金，加快提升传统服务业，壮大现代服务业，大力发展生产性和生活性服务业。

**改造提升商贸服务业。**大力发展连锁经营、仓储式超市、商业特许经营等新兴业态，改造提升传统商贸业。培育发展商贸流通骨干企业，力争年销售收入过亿元的企业达30家、过10亿元企业6家。支持迎江区建设省城市商贸服务业发展示范区。深入开展“农超对接”、“区超对接”，建立完善鲜活农产品供应链。进一步改造、提升和完善现有综合性市场，推进农贸市场回归公益性改革。大力推进城区商业设施转型升级。开发利用人防工程，建设大型地下商贸城。培育区域性再生资源回收体系。支持老字号与知名品牌餐饮业加快发展。

**大力发展现代物流业。**规划建设20个服务业集聚区，打造区域性物流中心。引进培育一批第三方、第四方物流品牌企业。大力发展城市配送物流、农村物流、区域物流和国际物流，扶持壮大一批龙头企业，建立完善现代物流体系。积极开发和应用物流先进技术，加快建立功能齐全的公共物流信息平台，加强物流标准化建设，促进信息资源共享和物流网络互联互通。大力推进皖江农产品、城西综合物流园等4个特色物流园区及一批配送中心和专业市场建设，在市区和8县（市）建设9个物流中心。加强口岸和通关建设，加快岸线资源整合和开发进程。

**着力壮大旅游业。**加快天柱山景区体制改革，寻求战略合作伙伴，发展旅游集团。强力推进百亿旅游项目工程，建成开放浮山、孔城老街一期、天峡、妙道山、仙龙湖等景区,加快天柱山旅游度假中心、孔雀东南飞文化园、司空山等项目建设,开工建设龙山凤水、潜山温泉、皖文化园、太湖梅河谷等重点项目。加强景区旅游公路等基础设施建设。强化宣传营销，拓展游轮、专列、直通车和大型团队市场。整合城区文化旅游资源，组建旅游集团公司。创建4A景区3个以上。

**加快培育新兴服务业。**围绕消费和产业结构升级，加快发展新兴服务业。为自主创新提供支撑，积极培育研发设计、科技孵化及咨询、创意服务、节能环保、信息软件等产业；为承接产业转移和集群发展提供配套，积极培育总部经济、会展经济、教育培训、人才中介、金融保险和服务外包；为提高人民幸福指数，积极培育体育健身、社区服务、物业管理、家政养老、休闲娱乐等生活性服务业。

**（六）强力推进示范区建设**

    今年是示范区建设关键之年。必须着眼于转型升级，突出加快产业培育、载体建设和环境优化，力促示范区建设取得实效。

**高水平承接产业转移。**坚持招大引强，提升产业承接规模和水平。围绕园区主导产业，加强承接关联配套企业，积极引进基地型和龙头型项目，推进集中连片开发。着力引进世界500强企业、大型央企和全国知名民企。坚持招才引智，引进国内外研发团队和高端人才，加快科技成果就地转化。引进一批符合产业发展方向、高附加值、低碳环保、拥有一定技术优势和核心竞争力的优势企业。

**加快园区转型升级。**明确各园区发展方向和战略重点，全面把好市场准入关、投资强度关和环境评价关，围绕主导产业和首位产业承接转移，优化园区产业结构。开展产业集中示范园区创建，加快中船配套产业园、中国（安庆）食品工业城等一批特色园区建设。大力推进园区合作共建，探索建立利益分享机制，创新园区建设管理模式。支持桐城申报建设国家级开发区。继续支持有条件的开发区升格为省级开发区。支持市化学工业区发展“绿色化工、生态园区、循环经济”，创建省级高新技术开发区。

**切实优化示范区发展环境。**充分运用先行先试权，完善示范区政策支持体系。构建多元平台，积极发展符合中小企业特点的投资基金，支持具有高成长性的中小企业申报发展集合债券和集合票据。加强职业教育和公共实训基地建设，融入皖江劳务合作，改善务工人员生活保障。积极创造条件，提高建设用地保障能力。

**（七）全力加强节能减排和环境保护**

加强环境保护是科学发展的刚性要求。必须把能源资源节约集约和环境保护摆上更加重要的位置，走低碳环保的新路子。

**强化节能减排。**推进建筑、交通运输、商业、公共机构等领域节能减排，突出抓好石油化工、建材、电力等重点耗能行业和年耗能3000吨标煤以上重点耗能企业节能降耗。强化主要污染物排放监控。严格新上项目节能评估，加大节能减排技术改造，淘汰落后产能，关闭高耗能、高污染企业，实行清洁生产审核，鼓励发展低能耗、低排放产业。继续加大重点行业环境执法。加快节能新技术、新工艺、新产品推广应用。着力提升县级污水、垃圾处理设施运行能力，加大重点建制镇环保设施投入，加强低碳燃烧、脱硝设施建设和管理。

**加强资源节约集约利用。**强化耕地保护，规范推进城乡建设用地增减挂钩试点，扎实推进农村土地整治。严格清理占而未用土地，支持旧城、旧镇、旧村改造，推进土地开发复垦整理，盘活存量土地。严格考核投资强度、产出率、建筑容积率、土地供应率等指标，节约集约利用土地。加强饮用水源区保护，确保居民饮水安全。

**推进循环经济发展。**探索建立能量回收体系，加强余热余压等资源综合利用，认真谋划资源综合利用项目。积极申报国家级循环经济示范园区、国家级再生资源试点市。争取部分园区和企业列入国家低碳试点。落实生产者责任延伸制度，建立循环经济评价指标体系，推动更多产业循环发展。

**（八）继续深化改革扩大开放**

改革开放是建设活力安庆的必由之路。必须牢牢把握加快改革创新这一强大动力，深化重点领域、关键环节改革，推进体制机制创新，为加快发展增添强大动力。

**深化重点领域改革。**全面深化国库集中收付制度改革，建立覆盖全市的预算执行动态监管体系；深化城区管理体制改革，进一步完善市区两级财税分配体制，合理界定城区经济社会管理权限；加快园区管理体制改革，积极推行园区市场化、公司化运营新机制；加快推进市直行政事业单位资产管理改革，将资产变成资本，在资本运营中实现国有资产保值增值，做强政府投融资平台；深化行政管理体制、基层医药卫生体制、文化体制和事业单位分类改革，建立适应市场经济要求的运行机制；大力推进农村金融创新，探索林权、承包经营权、住房和保单等抵押贷款的有效模式，广泛开辟农村融资渠道；大力发展非公有制经济，增强非公有制企业内生动力；实施企业一套表改革，提高统计数据质量。

**不断提升科技创新能力。**扎实推进国家技术创新工程试点，支持企业建立博士后工作站。争创国家级企业技术中心1户、省级企业技术中心4户以上，加强市级企业技术中心建设。建成桐城国家高分子材料质检中心。实施商标品牌战略，开展质量兴市、质量兴企活动，培育省级新产品、名牌和著名商标，鼓励企业以品牌为纽带进行并购重组。加快创新型企业建设，鼓励传统及优势产业大力实施科技创新，促进技术、人才等创新资源向优势企业聚集。推进与中科院等科研院所合作，深化与安庆师院校企合作。

**大力发展外向型经济。**推动出口结构升级，鼓励机电、高新技术、农副产品扩大出口。重点培育发展出口品牌，新增境外注册商标10个以上，企业和产品认证10个以上。加快纺织服装、高新科技、机械化工、农副产品、造船等出口基地建设，扩大自主知识产权、自主品牌、自主营销产品出口。推动对外承包工程与劳务合作。支持企业“走出去”，深化国际经济合作。加强进口工作，积极扩大进口，引导外资更多投向高端制造、节能环保等领域。

**（九）大力推进文化安庆建设**

    文化安庆赋予未来发展新的内涵。必须强力推进文化改革发展，加快实现由文化资源大市向文化强市跨越。

**繁荣发展文化事业。**实施文化民生工程，扎实推进乡镇综合文化站、农家书屋、广播电视村村通等公共文化设施建设，深入开展剧场、广场文艺演出、文化下基层进社区活动，发展新闻出版、广播影视、文学艺术事业。实施文艺精品工程，打造黄梅戏艺术创作研究基地，开展黄梅戏巡演活动，拓宽演出市场。精心办好第六届中国（安庆）黄梅戏艺术节。加快推进市博物馆、图书馆、文化馆、美术馆、独秀园二期工程建设。科学保护、合理利用各类文物和非物质文化遗产，开展宗教文化研究，加强历史文化名城名镇名村和街(区)保护。

**加快发展文化产业。**编制实施文化产业发展专项规划，建立文化产业发展基金。支持再芬黄梅公司建立现代企业制度，加快改制上市进程。加强文化企业家队伍建设。培育一批具有竞争力的骨干文化企业，组建市演艺集团。推进文化与科技融合，重点发展文化传媒、文化创意、演艺娱乐和文化会展等各类文化产业。大力发展黄梅戏特色演艺产业。加强历史文化资源开发利用，精心打造文化产业链。推进石牌戏曲文化产业园、邓石如碑林文化园等建设。积极开展对外文化交流，提升安庆文化传播力和竞争力。

**努力提高城市文化品质。**坚持以社会主义核心价值体系引领社会思潮，深入开展社会公德、职业道德、家庭美德、个人品德教育，加强和改进青少年思想道德建设；坚持用社会主义荣辱观引领社会风尚，提高群众文明素质，培育新时期市民精神，拓展公民道德实践；坚持以城市精神引领社会进步，强化“一个市民就是一扇窗口”意识，深入开展群众性精神文明创建活动，广泛开展“迎省运、讲文明、树新风”活动。以公共秩序、文明交通、市容市貌为重点，推动创建工作制度化、规范化、常态化，巩固省级文明城市创建成果，为争创全国文明城市奠定坚实基础。

**（十）切实加强以改善民生为重点的各项社会建设**

提高人民生活水平是全部工作的出发点和落脚点。必须牢牢把握保障改善民生这一根本目的，坚持以人为本，着力提高社会管理和公共服务水平，促进社会和谐稳定。

**拓展提升民生工程。**今年计划投入56亿元，实施42项民生工程。省定民生工程33项。市定民生工程9项，其中，被征地农民基本养老保险转入常态化管理；新增城市公交发展项目，尽快编制城市公交发展规划，推进公交线路延伸，填补公交线网空白点，改善居民乘车环境。新开工建设各类保障性住房2.12万套，逐步解决城镇低收入群众、新就业职工、农民工住房困难。建立稳定多元的筹资机制，加强民生工程项目管理和考核，探索创新项目管护机制，进一步提高已建成项目的使用效率，实现民生工程效益最大化。

**加强就业和社会保障。**重点做好高校毕业生、城镇就业困难人员和农民工就业，大力促进农村劳动力转移就业。加快建设统一规范灵活的人力资源市场，着力解决就业供需矛盾。加强社保和社会救助体系建设，加大对低收入群体帮扶救助，全面推进城乡医疗救助一站式服务，完善临时救助制度。保障粮油肉蛋菜等生活必需品市场供应，加强价格监测和监管，保持市场物价基本稳定。完善低保标准动态调整机制和困难群众物价补贴联动机制，切实保障特殊困难群体基本生活。继续实施残疾人康复救助工程。积极推进城乡养老保障体系建设。

**加快发展社会事业。**建立学前教育基金，实施学前教育三年行动计划。推进城乡义务教育学校标准化建设。推动普通高中多样化发展。大力发展职业教育。争取安庆技师学院早日获批，加快建设与发展。提升高等教育服务地方发展水平。巩固发展新型农村合作医疗制度,确保参合率稳定在98％以上。加强重大传染病防治工作。完善医疗卫生服务和基本药物供应保障体系。切实稳定低生育水平，综合治理出生人口性别比，大力提高出生人口素质。积极开展全民健身运动，办好市第十二届运动会，备战第十三届省运会。加强宗教事务管理，做好民族工作。加强县级国家综合档案馆建设。充分发挥工会、共青团、妇联等人民团体的作用。做好科普、地方志、外事、侨务、对台、老龄等工作。

**切实维护社会稳定。**深入推进社区综合管理体制改革，强化城乡社区自治和服务功能。加强流动人口和特殊人群管理服务，健全实有人口动态管理机制。落实企业安全生产主体责任，加强交通运输、建筑施工、消防、非煤矿山等重点领域安全隐患排查治理，严防重特大事故发生。认真实施“六五”普法规划。深入推进社会矛盾化解，严厉打击违法犯罪活动，加快建设覆盖城乡的社会治安防控体系。加强校园安全、校车安全、食品药品安全、医疗安全等工作。大力加强应急处置体系建设，切实抓好地震、地质、气象等灾害防治，提高保障公共安全和处置突发事件的能力。支持部队和后备力量建设。着力推进平安安庆建设，维护社会和谐稳定。

三、加强政府自身建设

加快“四个安庆”建设，深入实施“十二五”规划，对政府工作提出了新的更高要求。新一届县（市）区政府也面临新的使命。必须更加自觉地加强政府自身建设，切实提高科学发展水平。

**（一）强化责任意识，保持良好精神状态**

保持良好精神状态是责任政府履职尽责的重要前提。必须始终保持昂扬向上、奋发进取的良好状态。事业心和责任感决定工作姿态，彰显精神状态。要在解放思想上切实破除消极落后的观念束缚，敢闯敢干、大胆实践；在改革创新上不为任何困难和风险所阻碍，勇于担当、善于创造；在推进工作上说到做到、务求实效。切实以争创一流的志气、百折不挠的勇气、奋力开拓的锐气，咬定目标不动摇，真抓实干见成效。

**（二）强化法治意识，始终坚持依法行政**

 坚持依法行政是法治政府提高公信力的重要手段。必须强化依法行政意识，提高依法行政水平。坚持市政府常务会议学法制度。进一步清理、减少和调整行政审批事项。完善重大决策的调查研究、专家论证、群众评议制度，建立健全跟踪反馈和评估制度，全面推进重大决策和重点项目的社会稳定风险评估，优化行政决策程序，不断提高政府决策科学化、民主化水平。深化行政执法责任制，规范执法行为，强化执法监督，推进依法执法、公正执法、文明执法。实施行政强制法。加强行政执法队伍建设。自觉接受市人大及其常委会的工作监督、法律监督和市政协的民主监督，认真办理人大代表建议和政协委员提案。

**（三）强化为民意识，切实做好群众工作**

 坚持做好群众工作是为民政府密切干群关系的重要抓手。必须以人民满意为追求，强化为民意识，做到贴近群众、依靠群众、为了群众。推行服务质量公开承诺制，实行规范服务、便利服务。坚持调研工作制度。构建“大走访、下基层”活动长效机制。扎实推进网络问政常态化、制度化，拓宽民意表达渠道。创新群众工作方法，完善领导干部接访和工作联系点制度。重视做好来信来访工作，及时办理网友回复，引导群众理性合法地表达利益诉求，解决利益纠纷，依法保障群众利益。

**（四）强化效率意识，不断提升执行力**

 坚持提高行政效率是效能政府建设的重要追求。必须始终坚持效率至上，提速增效。深化“主要领导述效能、百名科长群众评”活动，实行职能部门主要领导年初公开承诺、年终公开述职，接受媒体、群众和社会监督。加强行政服务中心建设，强力推进“两集中、两到位”。创新政务公开方式，凡涉及群众利益和公共政策的事项，都要实行公开办理、限时办结。拓展电子政务范围，加强电子政务平台和服务中心建设。不断改进文风会风。完善政府绩效评价机制，加强目标管理，加大明察暗访力度，强化行政问责，严肃查处不作为、慢作为、乱作为，增强工作执行力。

**（五）强化廉洁意识，永葆人民公仆本色**

坚持清廉为民是廉洁政府执政宗旨的重要体现。必须恪守廉洁从政准则，做到为政不移公仆之心、用权不谋一己之私、立身不忘做人之本。落实党风廉政建设责任制，完善惩治和预防腐败体系，强化廉政风险防控。重点开展对加强和改善宏观调控、做好“三农”工作、保障和改善民生等政策措施落实情况的监督检查，确保政令畅通。进一步加大专项治理力度，严肃查办发生在工程建设、房地产开发、土地管理和矿产资源开发等领域的案件。加强政风行风建设，严肃查处损害群众利益、破坏发展环境的行为。严格控制“三公”经费开支，加强节约型机关建设。以廉洁之风推动发展、促进和谐、赢得民心。

各位代表！

今年是全面落实市第十次党代会精神的第一年，也是巩固“十二五”良好开局的关键一年，做好今年各项工作，意义尤为重大。让我们在中共安庆市委的坚强领导下，聚心聚力聚气，勤政廉政善政，开拓创新，扎实苦干，为“四个安庆”的美好明天而努力奋斗，以优异成绩迎接党的十八大胜利召开！
